# Optimal Inframammary Fold Incision Length Based on Implant Volume for Breast Enlargement: A Cadaveric Study

**Published:** 2019-03-19

**Authors:** Claude Muresan, Meghan M. Ford, Eric W. Anderson, Thomas J. Lee, Andrea R. Hiller, Swapnil D. Kachare, Bradon J. Wilhelmi

**Affiliations:** Division of Plastic Surgery, General Surgery Department, University of Louisville, Louisville, Ky

**Keywords:** breast augmentation, incision length, inframammary incision, implant volume, incision location

## Abstract

**Purpose:** Breast augmentation is the most commonly performed aesthetic operation in the Unites States annually. With the increasing popularity of gel implants, optimal incisional length for specific implant volumes becomes a factor to consider. Our study is the first, to date, to measure optimal incisional length for increasing Mentor smooth, round, moderate plus silicone implant volumes. **Method:** Three cadaver breasts were dissected in our anatomy laboratory. After dissection of a subpectoral pocket via an inframammary fold incision, time of implant insertion was measured for increasing volumes with the use of increasing incisional site lengths. **Results:** Values for increased incisions are as follows: 2.5-cm incision facilitated 100-cm^3^ implant (average time [AvgT] 76 seconds); 3-cm incision 100-cm^3^ implant (AvgT 32 seconds), 200 cm^3^ (AvgT 84.33 seconds); 3.5-cm incision 100-cm^3^ implant (AvgT 13.00 seconds), 200 cm^3^ (AvgT 22.00 seconds), 300 cm^3^ (AvgT 33.67 seconds); 4-cm incision 100-cm^3^ implant (AvgT 5.67 seconds), 200 cm^3^ (AvgT 11.33 seconds), 300 cm^3^ (AvgT 21.33 seconds), 400 cm^3^ (AvgT 26 seconds); 4.5-cm incision 100-cm^3^ implant (AvgT 5 seconds), 200 cm^3^ (AvgT 15.17 seconds), 300 cm^3^ (AvgT 19.67 seconds), 400 cm^3^ (AvgT 26 seconds), 500 cm^3^ (AvgT 39.67 seconds), 600 cm^3^ (AvgT 59.33 seconds), 700 cm^3^ (AvgT 78.67 seconds); 5-cm incision 100-cm^3^ implant (AvgT 1 second), 200 cm^3^ (AvgT 3.67 seconds), 300 cm^3^ (AvgT 8 seconds), 400 cm^3^ (AvgT 13 seconds), 500 cm^3^ (AvgT 19.33 seconds), 600 cm^3^ (AvgT 23.33 seconds), 700 cm^3^ (AvgT 28 seconds), 800 cm^3^ (AvgT 33 seconds). **Conclusion:** Based on our results, the optimal incision size for increasing Mentor smooth, round, moderate plus volumes is as follows: 2.5 cm for 100-cm^3^ implant, 3 cm for 200-cm^3^ implant, 3.5 cm for 300-cm^3^ implant, 4 cm for 400-cm^3^ implant, 4.5 cm for 500- to 700-cm^3^ implant, and 5 cm for 800-cm^3^ implant. This guideline can be used to provide the most aesthetic results without tissue compromise.

Approximately 300,000 breast augmentations are performed annually in the United States, making it the most commonly performed aesthetic operation.^1^ Tebett teaches 5 critical decisions in breast augmentation: pocket location, implant volume, implant type, optimal location for inframammary fold (IMF), and incision location. All these 5 aspects of augmentation mammoplasty have been extensively examined by authors in the literature.^2^^-^19 With the increasing popularity of gel implants, optimal incisional length for specific implant volumes becomes a factor to consider, which is not addressed in current literature.

A recurring theme seen in the literature is the description of techniques that allow placement of a concealed incision that concomitantly enables maximum access to the breast pocket.^2^^-^6^,^^9^^,^^11^ Papazian and colleagues^6^ describe a novel calculation of neo-IMF based on vertical implant dimension, whereas Lei and colleagues^17^ describe use of the Pythagorean theorem to aid optimal incision placement. Similar in each of these investigations is the use of the IMF incision, which remains the preferred approach for more than 64% of surgeons operating in the United states.^3^ This incision enables direct pocket visualization during implant placement, hemostatic control, and reoperation if needed.^8^^,^^9^ Some studies suggest the IMF incision is associated with lower rates of capsular contracture due to decreased seeding of skin flora on the implant.^7^^,^^10^^,^^11^ While there are numerous benefits to this incision, the downside of a noticeable scar is of primary importance to the patient seeking aesthetic surgery. This is most evident in a recent preoperative surgical survey of 216 Chinese women before undergoing augmentation mammoplasty. After being educated on the benefits and downsides of different incisional approaches, the majority (54%) of women in the study favored the use of an axillary approach because of a more concealed scar. When asked reasoning for choosing this incision, 44% of respondents said the most important factor in their decision was the visibility of the scar.^12^ Thus, it cannot be underemphasized that despite all technical advantages over an incision at the heart of all aesthetic patients is the ultimate scar, which, in their minds, will be used to determine in large part the success of the operation.

To this end, a short incision does not always equate to a more optimal final aesthetic result. Several factors need to be considered when selecting an appropriate incisional site length for a specific implant volume. Struggling to fit an implant into an inappropriately small incision can lead to tissue damage, stretch injury, and potentially skin necrosis. It also increases operative time and the risks of general anesthesia. More significantly, if not using a Keller funnel, an inappropriately small incision increases the implants contact to adjacent skin and thus the skin flora. This can potentiate capsular contraction by a subclinical microbial colonization of the implant.^6^ Capsular contraction remains one of the leading causes of patient dissatisfaction and ultimately costly reoperation. Therefore, adjuncts such as the Keller funnel and utilization of a “no-touch” technique have been adopted.^18^

Despite the key importance of the incisional length to both patient satisfaction and facilitating a technically efficient augmentation, there is a paucity of literature on this subject. We found no articles in the literature that specifically look at optimal incisional site lengths for specific gel implant volumes. Rather, the literature is filled with surgeon anecdotal recommendations for optimal incisional size, which seems to stem from personal experience or surgical dogma passed down during residency training from one generation to the next.^18^^,^^19^ Our study is the first, to date, that measures optimal IMF incisional lengths for increasing Mentor smooth, round silicone implant volumes.

## METHODS

**Figure 1:**
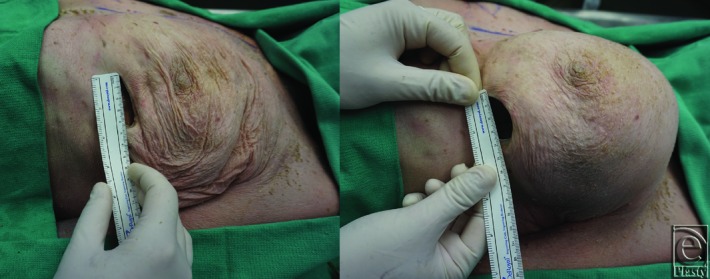
(Left) Preimplantation pocket dissection and measurement of a 4.5-cm inframammary incision. (Right) Incision size post–500-cm^3^ implant.

**Figure 2:**
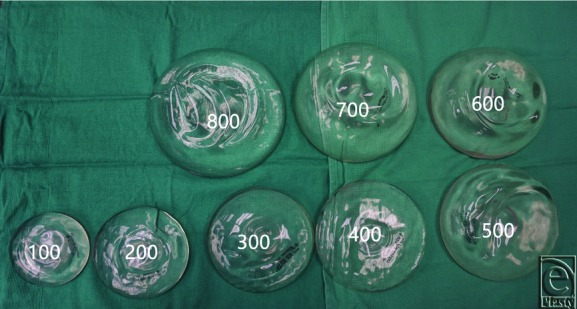
Mentor smooth, round, moderate profile plus silicone implants (100-800 cm^3^). Text numbers added to the photograph are the volume of implant in cubic centimeters.

Three cadaver breasts were dissected in our anatomy laboratory. Each dissection was carried out in similar fashion. First, a 2.5-cm IMF incision was made with a 10 blade scalpel. Next, using a combination of blunt and sharp dissections, a subpectoral pocket was created. Preimplantation incisional length was measured and recorded (Fig 1*a*). The amount of time required to fit a 100-cm^3^ Mentor smooth, round, moderate profile plus silicone implant into the mammary pocket was then recorded. The timer began immediately upon initiation of implant insertion and concluded once the implant was completely enclosed in the pocket. Post–incisional site length was then measured (Fig 1*b*). If the post–incisional site size increased or the amount of time for implant insertion was greater than 90 seconds, we concluded the preimplantation length to be too small for the given implant volume. Of note, the 90-second time mark was set to have a second objective measurement of determining experimental endpoint. We believe that operative time for augmentation mammoplasty procedures should not be increased significantly by implantation and thus being able to perform this portion of the operation should not exceed 1 minute and a half. If postimplantation incision size was increased or implantation took greater than 90 seconds, we subsequently increased incision length by half a centimeter and repeated the process. The experiment was carried out until all Mentor smooth, round, moderate profile plus implants from 100 to 800 cm^3^ were successfully implanted (Fig 2). Corresponding base diameters (Bds) and projection (P) in centimeters, for increasing implant volumes, are as follows: 100 cm^3^ 8.2, Bd 2.7 P; 200 cm^3^ 10.5, Bd 3.2 P; 300 cm^3^ 12.0, Bd 3.6 P; 400 cm^3^ 13.1, Bd 4.0 P; 500 cm^3^ 14.1, Bd 4.3 P; 600 cm^3^ 15.0, Bd 4.6 P; 700 cm^3^ 15.8, Bd 4.9 P; and 800 cm^3^ 16.5, 15.1 P.

## RESULTS

**Figure 3:**
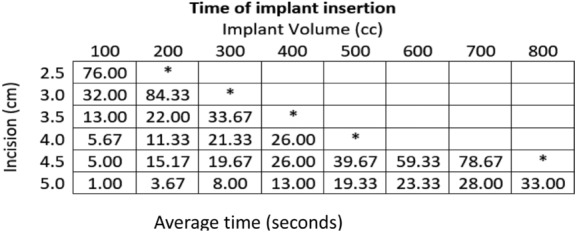
Time of implant insertion for a given implant volume based on incisional length. Average time (in seconds) of insertion of the implant based on incisional length (in centimeters). *Failed implantation due to postimplantation length or average time of more than 90 seconds.

**Figure 4:**
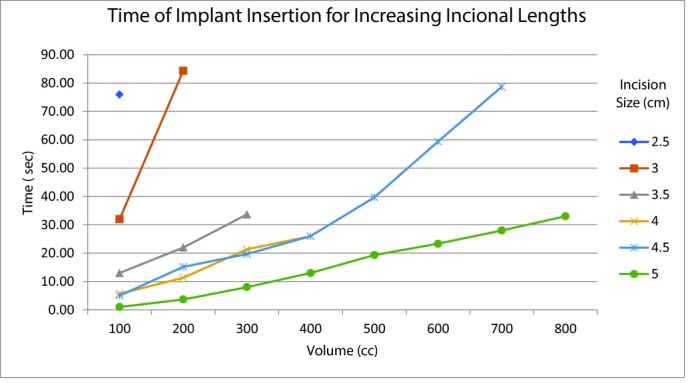
Time of implant insertion for a given implant volume based on incisional length.

Our results demonstrate that only the 100-cm^3^ implant could fit into the subpectoral pocket with a 2.5-cm incision without increasing the postimplantation incisional site length or going over the 90-second time mark. The average time (AvgT) over the 3 trials for implantation of the 100-cm^3^ implant using the 2.5-cm incision was 76 seconds. Average times, rounded to the nearest 100th second, for increased incisions, are as follows: 3-cm incision 100-cm^3^ implant (AvgT 32 seconds), 200 cm^3^ (AvgT 84.33 seconds); 3.5-cm incision 100-cm^3^ implant (AvgT 13.00 seconds), 200 cm^3^ (AvgT 22.00 seconds), 300 cm^3^ (AvgT 33.67 seconds); 4-cm incision 100-cm^3^ implant (AvgT 5.67 seconds), 200 cm^3^ (AvgT 11.33 seconds), 300 cm^3^ (AvgT 21.33 seconds), 400 cm^3^ (AvgT 26 seconds); 4.5-cm incision 100-cm^3^ implant (AvgT 5 seconds), 200 cm^3^ (AvgT 15.17 seconds), 300 cm^3^ (AvgT 19.67 seconds), 400 cm^3^ (AvgT 26 seconds), 500 cm^3^ (AvgT 39.67 seconds), 600 cm^3^ (AvgT 59.33 seconds), 700 cm^3^ (AvgT 78.67 seconds); 5-cm incision 100-cm^3^ implant (AvgT 1 second), 200 cm^3^ (AvgT 3.67 seconds), 300 cm^3^ (AvgT 8 seconds), 400 cm^3^ (AvgT 13 seconds), 500 cm^3^ (AvgT 19.33 seconds), 600 cm^3^ (AvgT 23.33 seconds), 700 cm^3^ (AvgT 28 seconds), 800 cm^3^ (AvgT 33 seconds) (Figs 3 and 4). Based on our results, the optimal incision size for increasing implant volumes is as follows: 2.5 cm for 100-cm^3^ implant, 3 cm for 200-cm^3^ implant, 3.5 cm for 300-cm^3^ implant, 4 cm for 400-cm^3^ implant, 4.5 cm for 500- to 700-cm^3^ implant, and 5 cm for 800-cm^3^ implant.

## DISCUSSION

Augmentation mammoplasty is the most common aesthetic operation performed in the United States annually.^1^ With such a high demand for this operation so are patient expectations for an aesthetically flawless final result. Given such pressures, the authors have described in detail many aspects of the operation: pocket location, implant volume, implant type, optimal location for IMF, and incision location.^2^^-^19 However, this is the first study that measures optimal incisional lengths for increasing gel implant volumes.

Current literature often states optimal incisional lengths for augmentation mammoplasty to vary in size from 4 to 6 cm. These numbers are quoted repeatedly throughout the literature and have become an accepted standard.^2^^,^^13^^,^^14^ However, there is a lack of any objective data to support these values. Furthermore, these incisional sizes are not specific to an implant volume, base diameter, or any other aspect of the case. Rather, they are intended to be used as universal incisional lengths for all different types, shapes, and sizes of implants. Given the importance the size of the incision has to aesthetic patients and to the surgeon in maximizing operative efficiency, we are surprised that this area is overlooked in current studies.

Both inappropriately small and conversely large incisions have negative implications on final aesthetic result. It is clear that an 800-cm^3^ implant will not readily fit into a 2.5-cm incision, and one does not require a study to poof this fact. However, trying to fit an 800-cm^3^ implant into an inappropriately small 4-cm incision leads to many negative downstream effects. The most direct being stretch injury causing local ischemia, tissue damage, and potentially skin necrosis. Struggling to fit an oversized implant into a small incision will also increase operative time, risks of general anesthesia, and overall costs. Direct microbial colonization of the implant from adjacent skin, which is hypothesized to be one of the leading causes of capsular contracture, is also increased by having too small an incision.^5^ Simply making an excessively large incision is not a valid solution. Aesthetic patients are innately tuned to every and any visible irregularity, most obvious being the final scar. Although having a larger incision may facilitate a technically easier, at times safer, and more rapid operation, it does so at the expense of a larger scar. The literature has demonstrated that final scar length is of monumental importance for aesthetic patients.^12^ Therefore, having an objective-based guideline for incisional site length is long overdue.

Our study is the first study, to date, that quantifies optimal incisional length for increasing Mentor gel implant volumes. In order to match tissue characteristics, including elasticity, to that of living patients, the study was carried out on fresh cadavers.^20^ The IMF incision was chosen because it provides the most direct access and clear visualization to the breast pocket.^8^^,^^9^ We chose to quantify incisional length to implant volume, as we believe volume determines in large part length of incision. Furthermore, associating a specific volume to an incision length is more readily recalled once a guideline is established. It should be noted that implant diameter was also considered as a possible reference to quantify incisional size length. However, because various sized implant volumes can have a spectrum of associated diameters, often being within a millimeter apart, we felt such small differences would not change the optimal incisional size. Furthermore, when inserting an implant, profile needs to be considered, which is better accomplished by using volumes. Based on our results, the optimal incision size for increasing implant volumes is as follows: 2.5 cm for 100-cm^3^ implant, 3 cm for 200-cm^3^ implant, 3.5 cm for 300-cm^3^ implant, 4 cm for 400-cm^3^ implant, 4.5 cm for 500- to 700-cm^3^ implant, and 5 cm for 800-cm^3^ implant.

Using objective measurements, our study is the first of its kind to provide specific incisional size recommendations based on implant volume. These guidelines were established by implantation of Mentor smooth, round, moderate profile plus silicone implants via an IMF incision. It should be stressed that the guidelines we have created are specific for Mentor smooth, round, moderate plus implants. Different implant manufactures as well as different Mentor types will have different profiles, base diameters, malleability, and cohesivity, therefore not allowing extrapolation of incisional size based solely on implant volume. Future studies can investigate whether changing the implant manufacture and type will affect incisional size. To this end, the addition of a Keller funnel will likely also affect the incisional length and is future investigation. Our study is only the first of potentially many different investigations that will help establish a set of recommended guidelines to optimize incisional length for specific implant volumes.

## CONCLUSION

Based on our results, the optimal incision size for increasing Mentor smooth, round, moderate plus profile volumes are as follows: 2.5 cm for 100-cm^3^ implant, 3 cm for 200-cm^3^ implant, 3.5 cm for 300-cm^3^ implant, 4 cm for 400-cm^3^ implant, 4.5 cm for 500- to 700-cm^3^ implant, and 5 cm for 800-cm^3^ implant. Towing the line between too small and too large an incision, we have taken the first step toward creating an implant-specific guideline for surgeons to use in order to provide the most aesthetic results without tissue compromise.
